# Oligonucleotide Microarrays Identified Potential Regulatory Genes Related to Early Outward Arterial Remodeling Induced by Tissue Plasminogen Activator

**DOI:** 10.3389/fphys.2019.00493

**Published:** 2019-04-30

**Authors:** Olga Plekhanova, Yelena Parfyonova, Irina Beloglazova, Bradford C. Berk, Vsevolod Tkachuk

**Affiliations:** ^1^Faculty of Medicine, Lomonosov Moscow State University, Moscow, Russia; ^2^National Medical Research Center of Cardiology, Moscow, Russia; ^3^Aab Cardiovascular Research Institute, University of Rochester Medical Center, Rochester, NY, United States

**Keywords:** tissue type plasminogen activator, outward vascular remodeling, microarray, vascular tone, arterial injury

## Abstract

Constrictive vascular remodeling limiting blood flow, as well as compensatory outward remodeling, has been observed in many cardiovascular diseases; however, the underlying mechanisms regulating the remodeling response of the vessels remain unclear. Plasminogen activators (PA) are involved in many of the processes of vascular remodeling. We have shown previously that increased levels of tissue-type PA (tPA) contributes to outward vascular remodeling. To elucidate the mechanisms involved in the induction of outward remodeling we characterized changes in the expression profiles of 8799 genes in injured rat carotid arteries 1 and 4 days after recombinant tPA treatment compared to vehicle. Periadventitial tPA significantly increased lumen size and vessel area, encompassed by the external elastic lamina, at both one and 4 days after treatment. Among 41 differentially expressed known genes 1 day after tPA application, five genes were involved in gene transcription, five genes were related to the regulation of vascular tone [for example, thromboxane A2 receptor (D32080) or non-selective-type endothelin receptor (S65355)], and eight genes were identified as participating in vascular innervation [for example, calpain (D14478) or neural cell adhesion molecule L1 (X59149)]. Four days after injury in tPA-treated arteries, four genes, regulating vascular tone, were differentially expressed. Thus, tPA promotes outward arterial remodeling after injury, at least in part, by regulating expression of genes in the vessel wall related to function of the nervous system and vascular tone.

## Introduction

Vascular remodeling is one of the most important mechanisms responsible for lumen narrowing in many cardiovascular pathologies (Paneni et al., 2017). Inward arterial remodeling is associated with high cardiovascular mortality, while outward vascular remodeling is considered to be a positive compensatory mechanism that provides adequate blood flow (van Varik et al., 2012). We have demonstrated previously that tissue-type plasminogen activator (tPA) attenuated inward arterial remodeling (Parfyonova et al., 2004).

Despite the active interest of many research groups to the problem of regulation of arterial remodeling, highly effective mechanisms are still to be found (Goel et al., 2012). The mechanisms, underlying tPA effects in the vessel wall, stay unclear.

Vascular remodeling involve many processes including cell proliferation, migration, extracellular matrix remodeling, and changes in vascular tone (Alexander and Owens, 2012; van Varik et al., 2012). There are contradictory data about the influence of tPA; it contributes to cell proliferation, but also in the previous study, tPA reduced neointima formation (Parfyonova et al., 2004).

To clarify mechanisms that may lead to the positive outward remodeling induced by tPA, we hypothesized that tPA might affect the expression of genes, which might regulate the remodeling response of the injured artery. To assess the role of gene expression changes in early processes leading to the outward vascular remodeling induced by tPA, we investigated alterations in gene expression profiles of rat common carotids after injury and local treatment with the recombinant tPA.

## Materials and Methods

### Ethics Statement

Animal studies were conducted in accordance with the principles of the Basel Declaration, approved guidelines of the Institutional Animal Care and Use Committee of the National Medical Research Center of Cardiology (Permit No. 385.06.2009) and the Guide for the Care and Use of Laboratory Animals (8th Edition, 2011, The National Academies Press, United States).

### Animal, Experimental Design, Surgery and Tissue Collection

Male Wistar-Kyoto rats (4–5 months old, weight 300–350 g) were obtained from a standard colony. Ketamine anesthesia was used (100 mg/kg body wt) and the balloon catheter (Fogarty 2F) injury of rats left common carotid arteries was performed. tPA (20 nmol/kg, Alteplase, Boehringer Ingelheim Pharma) or 500 μl of vehicle (saline) in Pluronic gel F-127 (BASF) was applied around injured carotids (Plekhanova et al., 2008). At days 1, 4, or 14 after injury the perfusion-fixation was done under anesthesia, the absence of endothelium in the injured area was verified by Evans blue dye (Gangadharan et al., 2001) and paraffin embedding of common carotids was performed (Plekhanova et al., 2000). For microarrays, 1 and 4 days after the operation carotids were rapidly removed and frozen in liquid nitrogen. Total RNA was extracted from left carotid arteries of injured, sham-operated and intact rats (nine animals per group). Microarray analysis was performed (Plekhanova et al., 2008).

### Morphometry

Toluidine blue staining of cross-sections (10 μm) with subsequent analysis using a Zeiss microscope coupled to a ProgRess-3008 camera (Kontron Elektronik) and PC with an UTHSCSA ImageTool, version 3.0 (San Antonio, TX, United States) (Plekhanova et al., 2000) were performed.

### Microarray Analysis

Analysis was performed as previously described (Korshunov et al., 2006; Plekhanova et al., 2008). Total RNA was isolated with the use of Qiagen RNAeasy Micro kit, the quality of RNA was confirmed. For each probe vessels from three rats were pooled; three hybridizations were done for each treatment group. Microarray analysis was performed at the University of Rochester Microarray Core Facility in accordance to Affymetrix recommendations using Affymetrix rat genome U34A oligonucleotide microarrays with 8799 known genes and expressed sequence tags (ESTs) (Santa Clara, CA, United States). Streptavidin phycoerythrin stain (SAPE, Molecular Probes) was used. Target hybridization was detected and quantified with a Gene Array Scanner (Hewlett Packard/Affymetrix). “Array performance” was assessed. Hybridization efficiency and sensitivity was controlled using control transcripts spiked into the hybridization cocktail. Inter-array variability was assessed by Microarray Analysis Suite 5.0. For data normalization global scaling with target intensity of 500 across all probe sets was used (Brooks et al., 2002). To lower the rates of overall errors and the estimated false detection the invariant set normalization method and the model-based expression indexes for perfect match (PM)-only arrays using DNA-Chip Analyzer (dChip) software package were applied (Li and Hung Wong, 2001). The “Present” percentage in all arrays was >40%. The percentage of “Array-outlier” in all arrays was not more than 5%, showing the absence of serious contamination or hybridization problems. To exclude “Absent” genes filtering was performed limiting the analyses to targets, which were “present” in more than 20% of arrays. A minimal inter-array variability (*SD* ≤ 2%) of the mean signal (with 5% of signals trimmed from both the high and low ends) was observed across the 4458 targets after the presence/absence filtering. Then to identify statistically significant changes in gene expression the significance analysis of microarrays (SAM) software was applied (Welle et al., 2002). Comparison with parametric ANOVA and Welch’s approximate *t*-test for non-equal variances were also performed (GeneSpring Software). To get very robust results we accepted as significant only genes verified by both analyses (Hoffmann et al., 2002).

### Software

Microarray Suite 5.0 was used with the default parameters. DNA-Chip Analyzer software was kindly provided by Drs. Cheng Li and Wing Hung Wong, Computational Biology Lab, Department of Biostatistics, Harvard School of Public Health. SAM was kindly provided by the Department of Statistics, Stanford University. GeneSpring 5.0 Software was from Silicon Genetics.

### Quantitative Polymerase Chain Reaction (qPCR)

Quantitative RT-PCR analyses were performed using ABI Prism 7900HT sequence detection system (Applied Biosystems). Double-stranded cDNA template preparation and purification were performed with Ambion MessageAmp aRNA kit. The qPCR primers and Master Mix from RT2 Real-Time Gene Expression Assay kits (SuperArray) were used. The effective hybridization was testified by the expression of 7 housekeeping genes including ribosomal proteins L18 (M20156), L18a (X14181) and S9 (X66370), alpha-tubulin (V01227), thymosin beta-4 (M34043), ubiquitin (D17296), beta-actin (V01217), which consistently gave positive signals through all samples analyzed.

### Statistical Analysis

Results are represented as mean ± SEM. Jandel SigmaStat (*t*-test or ANOVA for multiple comparisons to limit false discovery rate, as appropriate) was used for statistical analysis. *P* < 0.05 was considered as statistically significant.

## Results

### tPA and Vessel Remodeling

**FIGURE 1:**
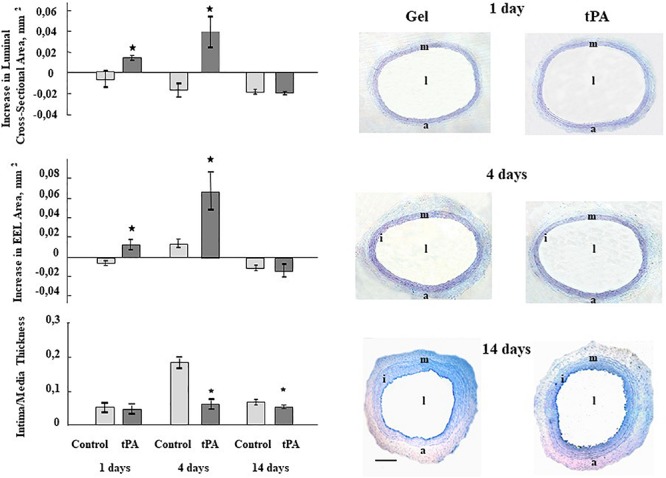
Bar graphs summarizing the effects of perivascularly applied recombinant tPA on luminal cross sectional area, external elastic laminae (EEL) area, and Intima-Media Thickness 1, 4, and 14 days after balloon injury to carotid arteries. Control represents balloon injured vessels, which were treated only with Pluronic gel/saline. For lumen, EEL and Intima-Media Thickness areas all injured vessels were compared with their corresponding right uninjured carotid artery in the same animals and the differences were calculated. Results are the means ± S.E.M of 6 to 7 animals in each group. *P < 0.05 from control. Histological figures depicting the effects of perivascular administration of recombinant tPA on the vessel structure 1, 4, and 14 days after balloon catheter injury of the carotid artery. l – lumen, i – intima, m –media, a – adventitia. Sections are stained with Toluidine Blue; magnification ×63. Scale bar 500 μm.

To assess tPA influence on the injured artery remodeling, we measured the area of lumen, the area, encompassed by the external elastic lamina (EEL), and intima-plus-media area [termed intima-media thickness (IMT)]; main parameters reflecting vessel remodeling. Periadventitial application of tPA increased EEL area (*P* < 0.05; Figure 1) at 1 day, then at 4 days there were significant increases in EEL and lumen areas, and a decrease in IMT (*P* < 0.05; Figure 1). Fourteen days after tPA treatment, there was still a decrease in IMT, but the other areas were the same (*P* < 0.05; Figure 1). These effects contrasted markedly with the effects of uPA that induced lumen narrowing, decreases in EEL and increased IMT after injury (Plekhanova et al., 2001, 2008). These data show that the arterial remodeling response stimulated by tPA induces lumen enlargement (Figure 1), increased EEL and decreased IMT, although the decrease in IMT only lasted 14 days.

### Differential Gene Expression Profiling Induced by tPA

To clarify the underlying mechanisms ensuring the vascular remodeling effects of tPA, the *in vivo* microarray analysis of gene expression was carried out. The expression of 8799 genes was investigated 1 and 4 days after the combination of balloon arterial injury plus treatment with recombinant tPA or control (gel plus saline).

One day after the operation in arteries treated with tPA the expressions of 95 transcripts (41 known genes, Supplementary Table 1A) were significantly different to those in control samples (*P* ≤ 0.05; confirmed by two statistical tests). Wherein amongst known genes 23 (56.1%) were down-regulated, when 18 (43.9%) were up-regulated after tPA treatment compared to control. At day 4, 53 mRNAs (28 known genes, Supplementary Table 1B) were significantly different in tPA group compared to gel-saline group. Among these known genes 5 (17.9%) were down-regulated and 23 (82.1%) were up-regulated compared to control.

In the group of genes differentially expressed after tPA treatment, a few groups of genes participating in the main processes of vascular remodeling including cellular migration, apoptosis and proliferation and inflammation were identified (Supplementary Tables 1A,B).

### tPA and Differential Expression of Nervous System – Related Genes

Among genes differentially expressed 1 day after injury and tPA treatment, eight genes were related to nervous system function (Table 1). Among these genes, seven were up-regulated, and one was down-regulated. At day four after injury the expression of one gene with the intended relevance to nervous system was increased in arteries treated with tPA. Changes in genes expressions of ≥1.5 fold were observed (Supplementary Figure 1). Thus, microarray gene analysis showed that tPA may contribute to the outward vascular remodeling at least partially through local changes in the expression of nervous system genes after arterial injury.

**TABLE 1:** Transcription profiles of genes related to nervous system and vascular tone regulation differentially expressed in rat carotid arteries treated with tPA compared to control vessels, which only received Pluronic gel/saline, 1 day and 4 days after balloon injury.

Nervous-system related genes				
Gene Identity	Genebank accession	Welch *t*-test P / direction		Effects relevant to Vascular Remodeling
**1 day**				
Calpain	D14478	0.02	up	anti-apoptotic
Substance P receptor (SPR)	M64236	0.03	up	pro-inflammatory, vasoactive
Neural cell adhesion molecule L1	X59149	0.02	up	pro-inflammatory
NMDA receptor glutamate-binding subunit	S61973	0.03	up	artery dilatation
Dihydropyrimidinase	D63704	0.03	up	unknown
Glycine transporter (GLYT-1)	U28975	0.03	up	unknown
Development-related protein Bdm1/NDRG4	AF045564	0.02	up	mitoch
Ataxin 3	Y12319	0.03	down	unknown
**4 days**				
Corticotropin releasing factor receptor	U53486	0.01	up	vasodilation

**Vascular tone regulation related genes**				

**1 day**				
RAC protein kinase alpha	D30040	0.02	up	anti-apoptotic; dependent on calpain
Calmodulin (pRCM1)	X13933	0.02	down	pro-proliferative
**4 days**				
Thromboxane A2 receptor	D32080	0.04	up	pro-inflammatory, pro-apoptotic
Corticotropin releasing factor receptor	U53486	0.01	up	nerv sys
Angiotensin II receptor type1	M90065	0.04	up	pro-proliferative
Non-selective-type endothelin receptor	S65355	0.04	down	pro-proliferative

### tPA – Induced Differential Regulation of Vascular Tone Related Genes

Genes involved in regulation of vascular tone were discovered to be differentially expressed in arteries both one and 4 days after injury and tPA application (Table 1). One day after injury and tPA treatment, one gene was up-regulated and one gene was down-regulated; while at 4 days after injury, three genes were up-regulated and 1 gene was down-regulated. Changes in genes expressions of 1.5–3.0 folds were observed (Supplementary Figure 2). These findings suggest the importance of tPA-mediated changes in vascular tone in vascular remodeling.

## Discussion

We demonstrated three major results regarding the role of tPA in vascular remodeling after injury. First, exogenous tPA significantly enlarged arterial lumen area at 1 as well as 4 days after balloon injury. Second, we discovered increases in EEL area at both one and 4 days. Finally, tPA decreased IMT at 4 and 14 days. These results show that tPA has a significant effect on vessel structure that appears to be beneficial. Our data are consistent with previous studies where local delivery of tPA via Dispatch catheters, followed by continuous intravenous infusion of tPA for 3 days, prevented intimal hyperplasia after angioplasty (Kanamasa et al., 2001). In contrast, an adenoviral construct expressing tPA enhanced neointima formation after angioplasty in a rabbit model (Hilfiker et al., 2001). Another study in cholesterol-fed rabbits showed that subcutaneous recombinant tPA inhibited the effect of balloon injury on luminal area compared with controls, but this effect did not reach statistical significance; the potential problem with the delivery method was discussed (Alexander et al., 2004). Taken together, these results indicate the importance of the model and the method used for tPA administration to evaluate its effects on arterial remodeling.

The gene array data shown here provide information regarding two likely mechanisms of action of tPA in the vessel wall: nerve function and vascular tone. Changes in nervous system–related gene expression after tPA treatment of injured arteries suggest that tPA-induced increases of vascular lumen size can be mediated at least in part through its effects on innervation that is consistent with the previous findings (Stenmark et al., 2011; Chistiakov et al., 2015). Some of these genes may affect vasodilatation. For example, NMDA receptor through nitric oxide (Faraci and Breese, 1993; Hama-Tomioka et al., 2012) or substance P receptor may elicit a neurogenic vasodilatation (Galligan et al., 1990; Charkoudian, 2010). Future work will be necessary to define precisely the roles of neurotransmitters in arterial remodeling. Of interest among the other nerve function genes, calpain was found to be highly expressed in the vasculature and is involved in many processes important for vascular remodeling (Letavernier et al., 2008). The involvement of its signaling in vascular remodeling regulation is extensively investigated nowadays (Kovacs and Su, 2017). Also smooth muscle cells neurotrophin-3 was shown to be implicated in vessel injury acting in an autocrine manner through its receptor TrkC (Donovan et al., 1995).

The microarray study also revealed in tPA-treated arteries changes in the expression of four genes related to vascular tone regulation, such as endothelin receptor or angiotensin receptor (Watanabe et al., 2005; Rapoport and Merkus, 2017). One of those genes - corticotrophin releasing factor receptor (U53486) is also involved in nervous system function. Considering that perivascular nerves may also affect vascular tone and induce vasodilatation (as well as tPA itself possesses vasoactive properties) (Heyman et al., 2004; Nassar et al., 2004), we may hypothesize that increases in lumen and EEL areas early after injury and tPA application may reflect changes in vascular tone, though it was not pronounced at the later stages due to the nature of vascular tone response.

The most novel finding here is the discovery that changes in genes related to nervous system function as well as vascular tone regulation, were highly significant at early time points, suggesting a possible connection between these two pathways leading to early outward vessel remodeling. There are many examples of relationships between nerves and vessels, especially regarding vascular tone, such as the release of vasoactive intestinal peptide by nerves to promote vasodilation in the gut.

In summary, the present data further support an important role for tPA as a negative regulator of IMT in response to balloon injury, as well as a mediator of favorable arterial remodeling. Furthermore, our genetic analysis reveal a novel role for the interaction between genes involved in the nervous system and those in vascular tone regulation suggesting new approach to limit IMT after injury.

## Ethics Statement

Animal studies were conducted in accordance with the principles of the Basel Declaration, approved guidelines of the Institutional Animal Care and Use Committee of the National Medical Research Center of Cardiology (Permit No. 385.06.2009) and the Guide for the Care and Use of Laboratory Animals (8th Edition, 2011, The National Academies Press, United States).

## Author Contributions

OP carried out the experiments and wrote the manuscript. YP and IB provided consultancy. BB supervised the project and wrote the manuscript. VT supervised the project.

## Conflict of Interest Statement

The authors declare that the research was conducted in the absence of any commercial or financial relationships that could be construed as a potential conflict of interest.

## References

[B1] Alexander B. Burnand K. G. Lattimer C. L. Humphries J. Gaffney P. J. Eastham D. (2004). The effect of anticoagulation with subcutaneously delivered polyethylene glycol conjugated hirudin and recombinant tissue plasminogen activator on recurrent stenosis in the rabbit double-balloon injury model. *Thromb. Res.* 113 155–161. 10.1016/j.thromres.2004.02.005 15115671

[B2] Alexander M. R. Owens G. K. (2012). Epigenetic control of smooth muscle cell differentiation and phenotypic switching in vascular development and disease. *Annu. Rev. Physiol.* 74 13–40. 10.1146/annurev-physiol-012110-142315 22017177

[B3] Brooks A. I. Stein C. S. Hughes S. M. Heth J. McCray P. M. Jr. Sauter S. L. (2002). Functional correction of established central nervous system deficits in an animal model of lysosomal storage disease with feline immunodeficiency virus-based vectors. *Proc. Natl. Acad. Sci. U.S.A.* 99 6216–6221. 10.1073/pnas.082011999 11959904 PMC122929

[B4] Charkoudian N. (2010). Mechanisms and modifiers of reflex induced cutaneous vasodilation and vasoconstriction in humans. *J. Appl. Physiol.* 109 1221–1228. 10.1152/japplphysiol.00298.2010 20448028 PMC2963327

[B5] Chistiakov D. A. Ashwell K. W. Orekhov A. N. Bobryshev Y. V. (2015). Innervation of the arterial wall and its modification in atherosclerosis. *Auton. Neurosci.* 193 7–11. 10.1016/j.autneu.2015.06.005 26164815

[B6] Donovan M. J. Miranda R. C. Kraemer R. McCaffrey T. A. Tessarollo L. Mahadeo D. (1995). Neurotrophin and neurotrophin receptors in vascular smooth muscle cells: regulation of expression in response to injury. *Am. J. Pathol.* 147 309–324. 7639328 PMC1869811

[B7] Faraci F. M. Breese K. R. (1993). Nitric oxide mediates vasodilatation in response to activation of N-methyl-D-aspartate receptors in brain. *Circ. Res.* 72 476–480. 10.1161/01.res.72.2.476 8380361

[B8] Galligan J. J. Jiang M. M. Shen K. Z. Surprenant A. (1990). Substance P mediates neurogenic vasodilatation in extrinsically denervated guinea-pig submucosal arterioles. *J. Physiol.* 420 267–280. 10.1113/jphysiol.1990.sp017911 1691291 PMC1190048

[B9] Gangadharan S. P. Eslami M. H. Weiss I. P. Sui X. Conte M. S. (2001). Monocyte adhesion to balloon-injured arteries: the influence of endothelial cell seeding. *J. Vasc. Surg.* 33 1247–1254. 10.1067/mva.2001.114211 11389425

[B10] Goel S. A. Guo L. W. Liu B. Kent K. C. (2012). Mechanisms of post-intervention arterial remodelling. *Cardiovasc. Res.* 96 363–371. 10.1093/cvr/cvs276 22918976 PMC3500049

[B11] Hama-Tomioka K. Kinoshita H. Nakahata K. Kondo T. Azma T. Kawahito S. (2012). Roles of neuronal nitric oxide synthase, oxidative stress, and propofol in N-methyl-D-aspartate-induced dilatation of cerebral arterioles. *Br. J. Anaesth.* 108 21–29. 10.1093/bja/aer368 22086508

[B12] Heyman S. N. Hanna Z. Nassar T. Shina A. Akkawi S. Goldfarb M. (2004). The fibrinolytic system attenuates vascular tone: effects of tissue plasminogen activator (tPA) and aminocaproic acid on renal microcirculation. *Br. J. Pharmacol.* 141 971–978. 10.1038/sj.bjp.0705714 14993107 PMC1574281

[B13] Hilfiker P. R. Waugh J. M. Li-Hawkins J. J. Kuo M. D. Yuksel E. Geske R. S. (2001). Enhancement of neointima formation with tissue-type plasminogen activator. *J. Vasc. Surg.* 33 821–828. 10.1067/mva.2001.112323 11296338

[B14] Hoffmann R. Seidl T. Dugas M. (2002). Profound effect of normalization on detection of differentially expressed genes in oligonucleotide microarray data analysis. *Genome Biol.* 3:RESEARCH0033. 10.1186/gb-2002-3-7-research0033 12184807 PMC126238

[B15] Kanamasa K. Inoue Y. Otani N. Naito N. Morii H. Ikeda A. (2001). tPA via infusion catheters followed by continuous IV infusion for 3 days prevents intimal hyperplasia after balloon injury. *Angiology* 52 819–825. 10.1177/000331970105201203 11775623

[B16] Korshunov V. Nikonenko T. Tkachuk V. Brooks A. I. Berk B. C. (2006). Interleukin-18 and macrophage migration inhibitory factor are associated with increased carotid intima–media thickening. *Arterioscler. Thromb. Vasc. Biol.* 26 295–300. 10.1161/01.ATV.0000196544.73761.82 16293799

[B17] Kovacs L. Su Y. (2017). Redox-dependent calpain signaling in airway and pulmonary vascular remodeling in COPD. *Adv. Exp. Med. Biol.* 967 139–160. 10.1007/978-3-319-63245-2_9 29047085 PMC7036267

[B18] Letavernier E. Perez J. Bellocq A. Mesnard L. de Castro Keller A. Haymann J. P. (2008). Targeting the calpain/calpastatin system as a new strategy to prevent cardiovascular remodeling in angiotensin II-induced hypertension. *Circ. Res.* 102 720–728. 10.1161/CIRCRESAHA.107.160077 18258859

[B19] Li C. Hung Wong W. (2001). Model-based analysis of oligonucleotide arrays: model validation, design issues and standard error application. *Genome Biol.* 2:RESEARCH0032. 10.1186/gb-2001-2-8-research0032 11532216 PMC55329

[B20] Nassar T. Akkawi S. Shina A. Haj-Yehia A. Bdeir K. Tarshis M. (2004). In vitro and in vivo effects of tPA and PAI-1 on blood vessel tone. *Blood* 103 897–902. 10.1182/blood-2003-05-1685 14512309

[B21] Paneni F. Diaz Cañestro C. Libby P. Lüscher T. F. Camici G. G. (2017). The aging cardiovascular system: understanding it at the cellular and clinical levels. *J. Am. Coll. Cardiol.* 69 1952–1967. 10.1016/j.jacc.2017.01.064 28408026

[B22] Parfyonova Y. Plekhanova O. Solomatina M. Naumov V. Bobik A. Berk B. C. (2004). Contrasting effects of urokinase and tissue-type plasminogen activators on neointima formation and vessel remodeling early after arterial injury. *J. Vasc. Res.* 41 268–276. 10.1159/000078825 15192267

[B23] Plekhanova O. Parfyonova Y. Bibilashvily R. Domogatskii S. Stepanova V. Gulba D. C. (2001). Urokinase plasminogen activator augments cell proliferation and neointima formation in injured arteries via proteolytic mechanisms. *Atherosclerosis* 159 297–306. 10.1016/S0021-9150(01)00511-1 11730809

[B24] Plekhanova O. S. Parfyonova Y. Bashtrykov P. P. Brooks A. I. Tkachuk V. Parfyonova Y. (2008). Oligonucleotide microarrays reveal regulated genes related to inward arterial remodeling induced by urokinase plasminogen activator. *J. Vasc. Res.* 46 177–187. 10.1159/000156703 18812699

[B25] Plekhanova O. S. Parfyonova Y. V. Bibilashvily R. S. Stepanova V. V. Erne P. Bobik A. (2000). Urokinase plasminogen activator enhances neointima growth and reduces lumen size in injured carotid arteries. *J. Hypertens.* 18 1065–1069. 10.1097/00004872-200018080-00011 10953998

[B26] Rapoport R. M. Merkus D. (2017). Endothelin-1 regulation of exercise-induced changes in flow: dynamic regulation of vascular tone. *Front. Pharmacol.* 8:517. 10.3389/fphar.2017.00517 29114220 PMC5660699

[B27] Stenmark K. R. Nozik-Grayck E. Gerasimovskaya E. Anwar A. Li M. Riddle S. (2011). The adventitia: essential role in pulmonary vascular remodeling. *Compr. Physiol.* 1 141–161. 10.1002/cphy.c090017 23737168 PMC4169049

[B28] van Varik B. J. Rennenberg R. J. Reutelingsperger C. P. Kroon A. A. de Leeuw P. W. Schurgers L. J. (2012). Mechanisms of arterial remodeling: lessons from genetic diseases. *Front. Genet.* 3:290. 10.3389/fgene.2012.00290 23248645 PMC3521155

[B29] Watanabe T. Barker T. A. Berk B. C. (2005). Angiotensin II and the endothelium: diverse signals and effects. *Hypertension* 45 163–194. 10.1161/01.HYP.0000153321.13792.b9 15630047

[B30] Welle S. Brooks A. I. Thornton C. A. (2002). Computational method for reducing variance with Affymetrix microarrays. *BMC Bioinformatics* 3:23. 10.1186/1471-2105-3-23 12204100 PMC126253

