# Supplementary figures and images for: Oligonucleotide Microarrays Identified Potential Regulatory Genes Related to Early Outward Arterial Remodeling Induced by Tissue Plasminogen Activator
Source: Front Physiol. 2019 Apr 30;10:493. doi: 10.3389/fphys.2019.00493 (PMC6502959; doi:10.3389/fphys.2019.00493)

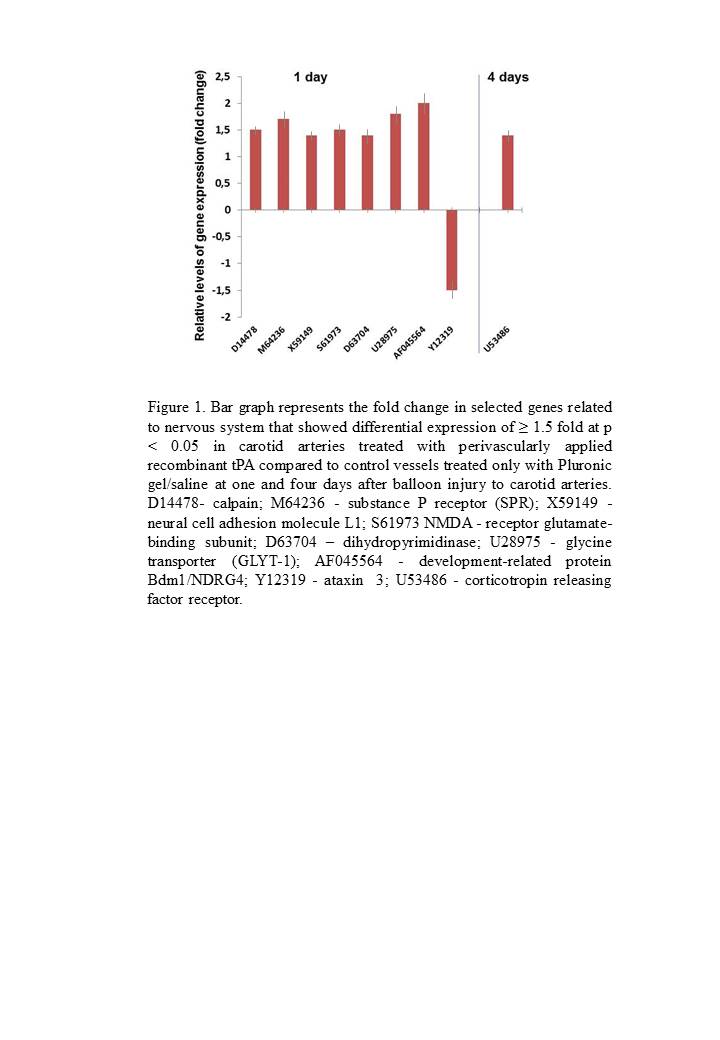

Supplement: Supplementary file 1 [file Image_1.jpg]

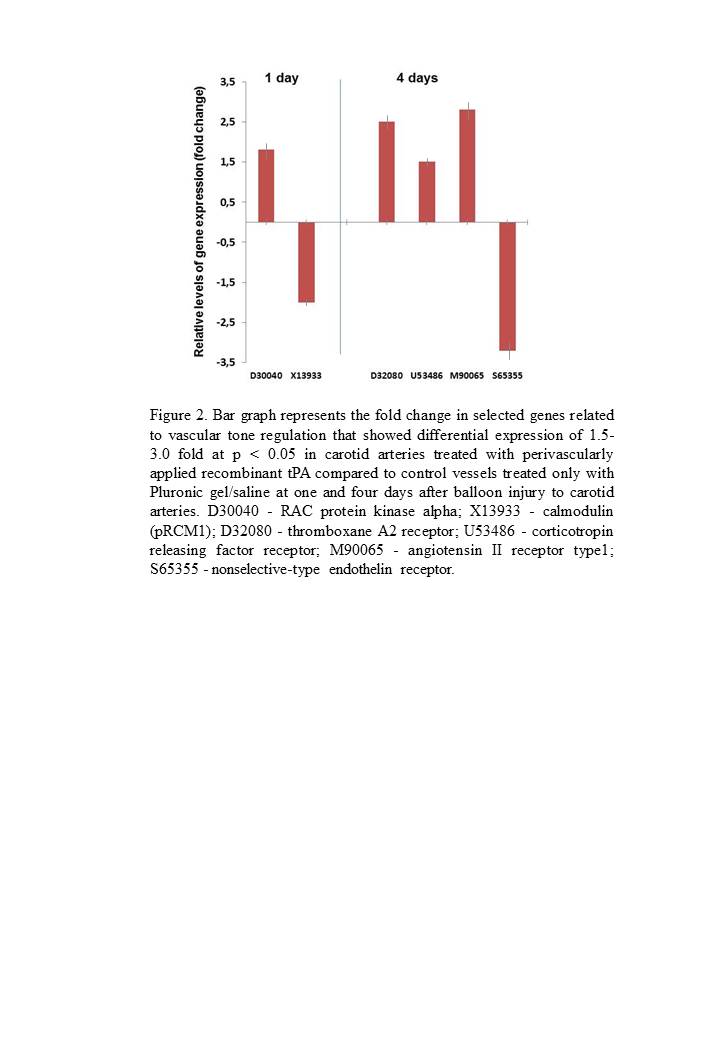

Supplement: Supplementary file 2 [file Image_2.jpg]
