# Supplementary material for: Oligonucleotide Microarrays Identified Potential Regulatory Genes Related to Early Outward Arterial Remodeling Induced by Tissue Plasminogen Activator
Source: Front Physiol. 2019 Apr 30;10:493. doi: 10.3389/fphys.2019.00493 (PMC6502959; doi:10.3389/fphys.2019.00493)
Supplement: Supplementary file 3 [file Table_1.docx]

**Table 1 A**. Transcription profiles of genes differentially expressed in rat carotid artery 1 day after balloon injury treated with tPA compared to control vessels, which only received Pluronic gel/saline.

| **Gene Identity** | **Genebank**  **accession** | **Welch t-test**  **P / direction** | | **Effects relevant to Vascular Remodeling** |
| --- | --- | --- | --- | --- |
| Migration & Cytoskeleton | | | | |
| acetylcholinesterase-associated collagen (COLQ) | AF007583 | 0.02 | up | unknown |
| calpain II 80 kDa subunit | L09120 | 0.03 | down | cytoskeletal reorganization |
| Tctex-1 | AB010119 | 0.04 | down | cytoskeletal reorganization |
| **Proliferation & Apoptosis** | | | | |
| calpain | D14478 | 0.02 | up | anti-apoptotic |
| AIM-1 (Ipl1/aurora kinase) | D89731 | 0.04 | down | mitotic regulator |
| Inflammation | | | | |
| substance P receptor (SPR) | M64236 | 0.03 | up | pro-inflammatory, vasoactive |
| neural cell adhesion molecule L1 | X59149 | 0.02 | up | pro-inflammatory |
| TNF-alpha converting enzyme (TACE) | AJ012603 | 0.02 | up | pro-inflammatory  pro-apoptotic |
| immunoglobulin rearranged gamma-chain | L07398 | 0.02 | up | pro-inflammatory |
| Transcription | | | | |
| epsilon 1 globin | X56326 | 0.03 | up | unknown |
| helicase (RAP30) | L01267 | 0.04 | down | unknown |
| zinc finger homeodomain enhancer-binding protein-1 (Zfhep-1) | U51583 | 0.04 | down | AT-2 receptor related |
| chromosomal protein HMG2 | D84418 | 0.04 | down | pro-inflammatory |
| cdc2 promoter | X60767 | 0.04 | down | pro-proliferative |
| Signaling | | | | |
| RAC protein kinase alpha | D30040 | 0.02 | up | anti-apoptotic;  dependent on calpain |
| NMDA receptor glutamate-binding subunit | S61973 | 0.03 | up | artery dilatation |
| dihydropyrimidinase | D63704 | 0.03 | up | unknown |
| protein phosphatase 2A (PP2A) 55 kD regulatory subunit alpha | M83298 | 0.02 | up | anti-apoptotic  pro-proliferative  pro-oxidant |
| calmodulin (pRCM1) | X13933 | 0.02 | down | pro-proliferative |
| flk protein-tyrosine kinase | X13412 | 0.04 | down | pro-migrative |
| S6 protein kinase | M58340 | 0.04 | down | pro-proliferative |
| leukocyte common antigen-related protein tyrosine phosphatase | L01702 | 0.04 | down | unknown |
| ADP-ribosylation factors like protein | U12402 | 0.04 | down | unknown |
| ADP-ribosylation factor-like protein 3 | U12568 | 0.04 | down | unknown |
| transient receptor potential plasma membrane channel (trp1 beta variant) | AF061266 | 0.04 | down | unknown |
| p58 | U44129 | 0.04 | down | cell survival |
| **Oxidation-related** | | | | |
| testosterone 6-beta-hydroxylase (CYP3A1) | L24207 | 0.02 | up | pro-oxidant |
| alcohol dehydrogenase 3 | X72792 | 0.02 | up | pro-oxidant |
| thiol-specific antioxidant (peroxiredoxin 5 ) | U06099 | 0.02 | down | anti-oxidant |
| **Metabolism & Others** | | | | |
| reggie-2 (Flotillin 2 ) | U60977 | 0.01 | up | unknown |
| putative pheromone receptor VN3 | U36895 | 0.05 | up | unknown |
| glycine transporter (GLYT-1) | U28975 | 0.03 | up | unknown |
| development-related protein Bdm1/NDRG4 | AF045564 | 0.02 | up | unknown |
| glycine methyltransferase | X06150 | 0.02 | up | unknown |
| 85kDa sialoglycoprotein (LGP85) | D10587 | 0.02 | down | unknown |
| ribosomal protein S13 | X53378 | 0.02 | down | pro-proliferative |
| ribosomal protein S25 | X62482 | 0.02 | down | unknown |
| ataxin 3 | Y12319 | 0.03 | down | unknown |
| alpha-propionyl-CoA carboxylase | M22631 | 0.03 | down | unknown |
| SC65 synaptonemal complex protein | X65454 | 0.04 | down | unknown |
| rPER2 (сlock gene) | AB016532 | 0.04 | down | unknown |

**Table 1 B.** Transcription profiles of genes differentially expressed in rat carotid artery 4 days after balloon injury treated with tPA compared to control vessels, which only received Pluronic gel/saline.

| **Gene Identity** | **Genebank**  **accession** | **Welch t-test**  **P / direction** | | | **Effects relevant to Vascular Remodeling** | |
| --- | --- | --- | --- | --- | --- | --- |
| Migration & Cytoskeleton | | | | | | |
| alpha-actin | J00692 | 0.04 | up | | smooth muscle phenotype | |
| H36-alpha7 integrin alpha chain | X65036 | 0.04 | up | | muscle fibers linkage system | |
| Inflammation | | | | | | |
| epoxide hydrolase | M26125 | 0.04 | up | | pro-inflammatory  pro-proliferative | |
| thromboxane A2 receptor | D32080 | 0.04 | up | | pro-inflammatory,  pro-apoptotic  vasoconstriction | |
| Signaling | | | | | | |
| fibromodulin | X82152 | 0.04 | up | | TGF inhibitor  attenuates neointima | |
| transforming growth factor beta-3 | U03491 | 0.03 | up | | pro-proliferative  pro-inflammatory | |
| angiotensin II receptor type1 | M90065 | 0.04 | up | | pro-proliferative | |
| ras-related protein (rad) | U12187 | 0.04 | up | | anti-migrative  attenuates neointima | |
| protein tyrosine phosphatase 2E (PTP2E) | U17971 | 0.04 | up | | unknown | |
| alternative brain Ca+2-ATPase | J04024 | 0.03 | up | | associated with proliferation | |
| ADP-ribosylation factor-like protein 4 | X77235 | 0.03 | up | | unknown | |
| phosphatidylinositol 5-phosphate 4-kinase gamma | AF030558 | 0.02 | up | | unknown | |
| aldose reductase | M60322 | 0.02 | up | | anti-proliferative | |
| FGF-9 | D14839 | 0.02 | up | | pro-proliferative | |
| adenylyl cyclase type V (GTPase activating protein) | M96159 | 0.01 | up | | unknown | |
| corticotropin releasing factor receptor | U53486 | 0.01 | up | | vasodilation | |
| lipocortin V (annexin) | AF051895 | 0.005 | down | | anti-inflammatory  anti-thrombotic  anti-apoptotic | |
| phospholipase C type IV | J05155 | 0.02 | down | | pro-proliferative  pro-migrative | |
| nonselective-type endothelin receptor | S65355 | 0.04 | down | | pro-proliferative  vasoconstriction | |
| **Oxidation-related** | | | | | | |
| flavin-containing monooxygenase 1 (FMO-1) | M84719 | 0.04 | up | | anti-oxidant | |
| **Metabolism & Others** | | | | | | |
| tyrosine-ester sulfotransferase | U32372 | 0.04 | up | | unknown | |
| RB109 (brain specific protein) | D26154 | 0.03 | | up | | unknown |
| neurotrophin-3 (HDNF/NT-3) | M34643 | 0.03 | up | | neuroprotection | |
| creatine transporter CHOT1 | X66494 | 0.03 | up | | unknown | |
| axonal glycoprotein (TAG-1) | M31725 | 0.02 | up | | unknown | |
| alpha-soluble NSF attachment protein | X89968 | 0.01 | up | | unknown | |
| serine proteinase rPC7 precursor | U36580 | 0.01 | down | | migration/inflammation? | |
| protein similar to preprotein translocase | U89745 | 0.01 | down | | unknown | |
